# Editorial: Obsessive-compulsive related disorders (OCRD) across the lifespan

**DOI:** 10.3389/fpsyt.2023.1296074

**Published:** 2023-10-06

**Authors:** Christine Lochner, Katarzyna Prochwicz, Edna Grünblatt

**Affiliations:** ^1^SAMRC Unit on Risk and Resilience in Mental Disorders, Department of Psychiatry, Faculty of Medicine and Health Sciences, Stellenbosch University, Cape Town, South Africa; ^2^Institute of Psychology, Faculty of Philosophy, Jagiellonian University, Kraków, Poland; ^3^Department of Child and Adolescent Psychiatry and Psychotherapy, Psychiatric University Hospital Zurich, University of Zurich, Zürich, Switzerland; ^4^Neuroscience Center Zurich, University of Zurich and the ETH Zurich, Zürich, Switzerland; ^5^Zurich Center for Integrative Human Physiology, University of Zurich, Zürich, Switzerland

**Keywords:** obsessive-compulsive and related disorders, lifespan, compulsive-impulsive, therapeutic alliance, family accommodation, eHealth, underpinnings, qualitative approach

## Introduction

Obsessive-compulsive and related disorders (OCRDs) constitute a group of psychiatric conditions characterized by obsessive thoughts and/or compulsive behaviors. In DSM-5, the section on OCRDs is narrowly defined, including obsessive-compulsive disorder (OCD), body dysmorphic disorder (BDD), hoarding disorder (HD), trichotillomania (TTM) or hair-pulling disorder, and skin-picking (excoriation) disorder (SPD) (1). The ICD-11 section on OCRDs is broader, also listing other conditions such as olfactory reference syndrome, hypochondriasis, and Tourette syndrome (2). A wider spectrum of compulsive-impulsive disorders, such as buying-shopping disorder and eating disorders, has also been proposed as OCRDs (3). While many of these have been described in the medical literature for some time, some have only recently been recognized, and others have yet to receive recognition in official nomenclatures.

While significant progress has been made in understanding these underrecognized disorders, there are still aspects not yet fully understood. This Research Topic in Frontiers in Psychiatry aimed to shed light on the current state of knowledge on OCRDs in general community and clinical samples of all ages. This Research Topic presents nine manuscripts, including a brief research report, a clinical trial, several original research papers, and a systematic review, from experts worldwide.

## Re OCD

Strappini et al. investigated the therapeutic alliance as a component of treatment in OCD. Their systematic review and meta-analysis provided evidence of an interactive effect between the therapeutic alliance and outcomes of cognitive behavior therapy (CBT) in patients with OCD. They recommended that future studies should focus on refinement of the temporal assessment of the alliance, in larger samples, and measurement of other interacting variables, to enrich the current understanding of therapeutic change factors that could benefit evidence-based treatment.

In addition to the therapeutic relationship, the relationship between OCD and family dynamics has also attracted increasing attention from researchers. The concept of family accommodation (FA) refers to family members' participation in patients' rituals and accommodating their compulsions to alleviate their anxiety. Addressing FA is an important part of the treatment plan. Liao et al. provided evidence of satisfactory psychometric properties of the Family Accommodation Scale Self-rated version (FAS-SR) in a large group of patients with OCD and relatives, suggesting that this scale can assist in the evaluation and treatment of OCD.

CBT including exposure exercises with response prevention (E/RP), is the established treatment of choice for OCD. However, there are many barriers to accessing CBT, including lack of availability, lack of experience of service providers, and financial and time constraints. Internet-based psychotherapy could help overcome some of these barriers and Hollmann et al. demonstrated the effectiveness of internet-based CBT in German children and adolescents with mild to moderate OCD.

Changing direction from investigations of treatment to the mechanisms underpinning OCD, Wang et al. explored the moderating effect of executive functioning on the relationship between anxiety and compulsive checking in adults with OCD. The study suggested that anxiety symptoms play a negligible role in explaining compulsive checking in individuals with OCD with relatively strong visuospatial working memory ability, but a substantial role in explaining compulsive checking in individuals with relatively weak visuospatial working memory. These findings encourage further research regarding how cognitive vulnerability factors of OCD and emotional factors interact to induce or maintain different OCD symptoms, providing additional insights into the mechanisms underpinning OCD.

## Re BDD

Addressing the paucity of research on, and low treatment rates of, mental illnesses in men, Kang et al. used survey to investigate BDD and depression in male university students in Malaysia. The study found that a significant proportion exhibited symptoms of BDD and depression, with BDD concerns mainly related to dissatisfaction with their height, which significantly correlated with the severity of depressive symptoms. This study responds to the quest for more research on the epidemiology of mental disorders among male adolescents, and highlights the importance of better support services.

In another contribution to this Research Topic on OCRDs, Brennan et al. used a qualitative approach to explore the lived experiences of individuals with BDD (In press). Three themes were identified: being consumed by the disorder, the *flawed self*, and intolerance of uncertainty about appearance, and were discussed in relation to the cognitive-behavioral model.

## Re compulsive buying/shopping

Aquino and Lins investigated the association between problematic buying/shopping with the Big Five's personality factors, during the COVID-19 pandemic, using an online survey of Portuguese adults. Regression analysis revealed significant correlations between various instances of buying/shopping, such as impulsive buying, compulsive buying, and panic buying, and some of the Big Five traits, highlighting the potential for improved understanding of these traits to inform preventive measures and effective treatment approaches.

Rocha et al. conducted a survey on the association between early maladaptive schemas (EMS) and compulsive and impulsive buying in young adults from Portugal. They found the *overvigilance* and inhibition schema was the main predictor of both impulsive and compulsive buying and that impaired limits was negatively associated with these tendencies. Coping mechanisms within this context were also explored.

Both aforementioned studies contribute to the conceptualization and study of problematic buying/shopping tendencies in populations other than Western, Educated, Industrialized, Rich, and Democratic (WEIRD) samples.

## Re OCRDs in general

Di Ponzio et al. reported on the positive effects of high-frequency repetitive transcranial magnetic stimulation (rTMS) over the left DLPFC in Italian individuals with OCRDs, including SPD, TTM and HD. This study suggests that this rTMS protocol is a promising treatment option for OCRDs and highlight common circuits involved in these disorders.

## Conclusion

In conclusion, the manuscripts included under this Research Topic cover a diverse range of themes, from the mechanisms underlying OCRDs, to the quest for personalized treatment approaches across the lifespan, while also addressing the misconception that these disorders are merely bad habits. The authors emphasized the need to further elucidate the etiologies and lifespan trajectories of OCRDs, as well as the development of eHealth treatment approaches. Nonetheless, this initiative demonstrates the progress made through global efforts, employing rigorous methodological standards, which can enhance clinical practice in addressing the unmet needs of patients throughout their lifetime.

## Author contributions

CL: Writing—original draft, Writing—review and editing. KP: Writing—review and editing. EG: Writing—review and editing.
